# A Hybrid Approach for Predicting PM_2.5_ Exposure

**DOI:** 10.1289/ehp.1002706

**Published:** 2010-10

**Authors:** Naresh Kumar

**Affiliations:** University of Iowa, Iowa City, Iowa, E-mail: naresh-kumar@uiowa.edu

van Donkelaar et al. (2010) integrated the satellite-based aerosol optical depth (AOD) and the chemical transport models (CTM) to develop concentrations of particulate matter < 2.5 μm in aerodynamic diameter (PM_2.5_). Because spatiotemporal coverage of *in situ* air pollution monitoring is limited, the integration of AODs with CTM is the wave of the future for developing time–space (and potentially source) resolved estimates of air quality. However, these methodologies have inherent limitations that the authors failed to address. van Donkelaar et al. (2010) based their research on work of Liu et al. (2004, 2007), but later research from the same authors (Paciorek and Liu 2009) acknowledged the limitations of Liu et al.’s earlier research. van Donkelaar et al. (2010) cited this research but did not address these limitations.

van Donkelaar et al. (2010) conceptualized that PM_2.5_ = η × AODs, where η is influenced by relative humidity (≥ 35 and ≥ 50% for North America and Europe, respectively) and computed using AOD_c_, the AOD from three-dimensional chemical transport models (3-D CTM). This has several problems: Failing to account for other factors, including boundary layer height, atmospheric pressure, and surface characteristics, can bias PM_2.5_ prediction. van Donkelaar et al. computed η at 2° × 2.5° and then interpolated η at 0.1° × 0.1°, which must have resulted in the same value of η for all 10 km AODs within each 2° × 2.5°area (at the equator), and hence strong spatial autocorrelation in the predicted PM_2.5_. Because the average lifetime of aerosols is one week and aerosols move across geographic space and time, AODs (i.e., the extinction of beam power due to the presence of aerosols) records a very strong spatiotemporal structure. Failing to account for spatiotemporal structure in AODs is likely to produce biased estimates of PM_2.5_ (Kumar 2010).

The CTM is a data-driven methodology, and the robustness of its output is largely dictated by input emission and meteorological data. Because such data are rarely complete and 100% accurate, it is difficult to accurately predict PM_2.5_ and AOD_c_ using CTM. Researchers are moving toward data assimilation techniques, in which predicted values are calibrated with respect to *in situ* measurements. van Donkelaar et al. failed to take advantage of data assimilation techniques to calibrate AODc.

Because of problems with version 5.0 or earlier of AODs (Levy et al. 2007), NASA is developing a Deep Blue version to estimate AODs over bright surfaces (Hsu 2010). Given the methodological constraints described above, I question van Donkelaar et al.’s (2010) conclusions. In their figures, the predicted PM_2.5_ in sub-Saharan Africa was unexpectedly high. It is unclear how coarse dust in that part of the world could result in high PM_2.5_ concentrations. This must be a result of the overestimated AODs due to surface brightness

The integration of AODs and CTM, coupled with spatiotemporal dynamic modeling, holds great potential to develop time–space resolved estimates of PM. Future research should be geared toward assimilation of the strengths of these methodologies. CTM has a great temporal resolution and is not constrained by cloud cover or biased by surface brightness, but the reliability of CTM output is dictated by the quality of input data. AODs have great spatial resolution (10 km) and can be estimated at finer spatial resolutions (5 km and 2 km), which is likely to be more robust than the coarse resolution AOD (Kumar et al. 2007); however, under cloud-free conditions it captures only two snapshots (at ~ 1030 hours and ~ 1330 hours local overpass time of the Terra and Aqua satellites) per day. Calibrating AODs for the problems mentioned above, daily (morning and afternoon) AODs can be produced globally. The best approach to integrating the strengths of these two methodologies would be to *a*) develop an empirical relationship between the calibrated AODs and AOD_c_ (estimated using a nested grid at a fine spatial resolution); *b*) utilize this relationship to predict a calibrated AOD_c_ (ÂOD_c_) for all data points with available AOD_c_; *c*) utilize ÂOD_c_ to predict PM_2.5c_ concentrations; *d*) develop an empirical relationship between predicted PM_2.5c_ and *in situ* measurements of PM_2.5_ with the adequate control for spatiotemporal structures and other subsidiary variables; and *e*) utilize this empirical relationship to develop the calibrated ~PM_2.5c_ (PM_2.5c_ predicted using the the empirical model) for all data points for which PM_2.5c_ is available. ~PM_2.5c_ in turn, can be aggregated and/or interpolated to any spatiotemporal scales using time–space Kriging, an interpolation method that minimizes error in the predicted values across geographic space and time.

## References

[b1-ehp-118-a425] Hsu NC (2010). Long-Term Global Aerosol Data Sets: Using Deep Blue to Synergize SeaWiFS, MODIS, and VIIRS Observations[Abstract].

[b2-ehp-118-a425] Kumar N (2010). What can affect AOD–PM_2.5_ association?. Environ Health Perspect.

[b3-ehp-118-a425] Kumar N, Chu AD, Foster A (2007). An empirical relationship between PM_2.5_ and aerosol optical depth in Delhi Metropolitan. Atmos Environ.

[b4-ehp-118-a425] Levy RC, Remer LA, Mattoo S, Vermote EF, Kaufman YJ (2007). Second-generation operational algorithm: retrieval of aerosol properties over land from inversion of Moderate Resolution Imaging Spectroradiometer spectral reflectance. J Geophys Res.

[b5-ehp-118-a425] Liu Y, Koutrakis P, Kahn R, Turquety S, Yantosca RM (2007). Estimating fine particulate matter component concentrations and size distributions using satellite-retrieved fractional aerosol optical depth: part 2—a case study. J Air Waste Manag Assoc.

[b6-ehp-118-a425] Liu Y, Park RJ, Jacob DJ, Li QB, Kilaru V, Sarnat JA (2004). Mapping annual mean ground-level PM_2.5_ concentrations using Multiangle Imaging Spectroradiometer aerosol optical thickness over the contiguous United States. J Geophys Res Atmos.

[b7-ehp-118-a425] Paciorek CJ, Liu Y (2009). Limitations of remotely sensed aerosol as a spatial proxy for fine particulate matter. Environ Health Perspect.

[b8-ehp-118-a425] van Donkelaar A, Martin RV, Brauer M, Kahn R, Levy R, Verduzco C (2010). Global estimates of ambient fine particulate matter concentrations from satellite-based aerosol optical depth: development and application. Environ Health Perspect.

